# Child and adolescent patterns of commuting to school

**DOI:** 10.1016/j.pmedr.2023.102404

**Published:** 2023-09-17

**Authors:** Claire Power, Patricia Fitzpatrick

**Affiliations:** UCD School of Public Health, Physiotherapy and Sports Science, University College Dublin, Belfield Dublin 4, Ireland

**Keywords:** Active transport, Active travel, Active travel to school, Active commuting, Physical activity, Physical activity and health

## Abstract

The World Health Organization stipulate children and adolescents should accumulate 60 min of physical activity (PA) daily; globally only 25% achieve this. Active travel to school (ATS) is a method of integrating PA into daily life with a documented health benefit accruing. Understanding factors associated with ATS is essential to inform a systems approach to increase ATS participation. This study described patterns of commuting to school and examined factors associated with ATS.

Children’s Sport Participation & Physical Activity Study 2018 data was used, an all-Ireland cross-sectional study of 6,650 students. Logistic regression analysis was performed to determine factors independently associated with ATS.

Most common commute to school methods were private car for primary (57%) and public transport for secondary (39%) students. The recommended 60 min of daily PA a week prior to the survey was achieved by 19.5% for primary and 12.6% for secondary students. Republic of Ireland (ROI) nationality (OR 1.09 95 %CI 1.02–1.16), meeting PA guidelines (OR 1.26 95 %CI 1.08–1.46), attending a ROI school (OR 2.27 95 %CI 2.02–2.57), attending a non-Delivering Equality of Opportunity in Schools (DEIS) school (OR 2.47 95 %CI 1.87–3.24), attending an urban school (OR 3.96 95 %CI 3.41–4.59) were each independently statistically significantly associated with ATS. Living in a family with a car (OR 0.27 95 %CI 0.19–0.39), attending secondary school (OR 0.69 95 %CI 0.62–0.78), attending a small sized (<33rd percentile) school (OR 0.68 95 %CI 0.60–0.77), living >5 km from school (OR 0.22 95 %CI 0.2–0.24) were each significantly negatively associated with ATS.

ATS is a means of increasing youth PA and health. Factors associated with ATS can inform further research and intervention to increase ATS participation.

## Highlights

• WHO state children & adolescents should accumulate 60 min of physical activity(PA)daily;globally only 25% achieve this.
• Active travel to school is a method of integrating PA into daily life with a documented health benefit accruing.
• This cross-sectional study included 1,548 primary and 5,102 secondary students.
• This study described patterns of commuting to school and examined factors associated with active travel to school.
• This study’s findings can inform a multi-sector systems approach to increase active travel to school.

## 1 Introduction

Physical activity (PA) is movement produced by skeletal muscle which utilises more energy than rest and is health augmenting. One can be very physically active without participation in formal sport or exercise. (Caspersen and Powell, 1985) Active commuting is fully or partially completing everyday journeys by foot or by bike, thus it is one form of PA. (Honda et al., 2022, Page et al., 2023).

The World Health Organization stipulate children and adolescents should accumulate 60 min of moderate-to-vigorous PA daily, include activities that strengthen muscle and bone development at least 3 times per week, and limit sedentary time. (World Health Organization, 2020) Greater than 80% of the world's adolescent population is insufficiently physically active and only 1 in 4 children meet recommended PA guidelines. (World Health Organization, 2020) In 2018 only 13% of children met PA guidelines across the island of Ireland. (Woods et al., 2018) The proportion of the population meeting PA guidelines in the Republic of Ireland (ROI) is documented in Table 1.

**Table 1:** 2021 Proportion of Population Meeting Physical Activity Guidelines in ROI (World Health Organization, 2021).

Age	Total	Male	Female
6–12 years	17 %	23 %	13 %
13–18 years	10 %	14 %	7 %
>18 years	46 %	54 %	38 %

Children living in Northern Ireland (NI) are least likely to meet the recommended levels of PA, when compared with other countries across the UK (Griffiths et al., 2013) and in Europe. (Van Hecke et al., 2016).

The 2022 Active Healthy Kids Global Alliance Ireland North and South PA report card found that active transportation participation decreased in ROI and NI since 2016. Findings also identified inequalities in PA participation, with girls, younger children and children from lower socioeconomic backgrounds being less likely to meet PA guidelines for health (Active Healthy Kids, 2022, The Institute of Public Health, 2022).

Physical inactivity is now recognised as an important public health issue and is closely tied to the obesity epidemic and to an ongoing rise in non-communicable disease. (World Health Organization, 2020) In past eras a necessity to commute actively allowed for routine daily PA. (Rütten et al., 2013) Children and adolescents aged ≥4 years have a daily requirement to travel from home to school. Increasing participation in active travel to school (ATS) offers an opportunity to increase PA in this cohort.

Kek et al. highlight that walking and/or cycling to school/work is a method of integrating PA into daily life and is associated with high levels of energy expenditure and health. (Kek et al., 2019) Forming healthy habits from a young age, for example ATS, is more optimal than trying to create such a habit later in life. (Kek et al., 2019) Those who report strong habits for active travel as children are more likely to actively commute as adults. (Panter et al., 2011) Interventions designed to encourage ATS can allow for active commuting to continue into adulthood. (Panter et al., 2011).

A scoping review of 25 child friendly policies and 19 sustainable travel policies by Gilber et al. (2018) found that research, policy, media and public health activity all tend to focus on adults being active in their daily commute. Overlooking youth has resulted in a steady decline in ATS in many countries. (Reilly and Tremblay, 2020) Reilly and Tremblay highlight that climate change and pandemic concern have provided an impetus to instil active lifestyle habits in children. (Reilly and Tremblay, 2020) Understanding factors associated with ATS is essential for public health planning and action to increase ATS participation. (Murtagh et al., 2016) This study aimed to describe the commuting to school patterns of children and adolescents in Ireland and to examine factors associated with ATS using data from the Children’s Sport Participation and Physical Activity Study (CSPPA).

## 2 Methods

### 2.1 Study population

This study was conducted using secondary data from CSPPA 2018. CSPPA 2018 was a cross-sectional study. The study population included 6,650 student respondents who completed the questionnaire in school settings. CSPPA 2018 is a follow up to CSPPA 2010 looking at participation in sport, PA and physical education among children and adolescents ≥10 years in ROI and NI. CSPPA 2018 was the first study to assess these issues in an all-island of Ireland context. It was a multi-centre study undertaken by the University of Limerick (lead university), Dublin City University, University College Cork and Ulster University. It was funded by Sport Ireland, Healthy Ireland and Sport Northern Ireland. (Irish Social Science Data Archive, 2020).

### 2.2 Sampling strategy

The CSPPA 2010 sampling strategy was followed for CSPPA 2018. CSPPA 2010 obtained a representative sample of youth in Ireland aged 10–18 years using a systematic, one-stage cluster sampling method. Schools were stratified by sex (male, female, mixed), geographic location (urban, rural by population density), socioeconomic status (designated or non-designated disadvantaged), type (primary school, secondary school, community college, comprehensive college, vocational school), size and fee paying status (fee or non-fee paying). Schools in areas with a population ≥1500 are designated urban and schools in areas with a population ≤1500 are designated rural in line with Census definitions. (CSO, 2019) ROI schools in disadvantaged areas can be assigned delivering equality of opportunity in schools (DEIS) status, qualifying them for additional supports from the Department of Education in an effort to promote equity in education. (Irish Department of Education, 2020) In ROI DEIS status was used to designate schools as disadvantaged. In NI the percentage of free meals distributed as low, medium or high was used to designate disadvantage. School size categorisation as small, medium or large was based on the total number of pupils attending. In ROI, school sizes were determined by creating tertiles (small: <33rd, medium: 33rd–66th, large: >66th percentile) using primary or post primary school pupil numbers, and for NI, the school size was supplied by the Department of Education NI. (Woods et al., 2018).

All 2010 schools (primary, N = 3292; secondary, N = 732), with the exception of special schools, within ROI were eligible for CSPPA 2018 sampling. Once a school was selected, every child ≥10 years in the specific year group for that school was deemed eligible and invited to participate; primary years were 5th and 6th class in ROI and year 6 and 7 in NI and secondary years were 1st to 6th in ROI and year 8 to 14 in NI. This method was feasible and used to maximise the likelihood of each cluster selected being a small-scale representation of the total population of that geographical area. CSPPA 2018 data were weighted to avoid bias from the clustered data. For the ROI CSPPA 2018 sample, 114 of the 123 CSPPA 2010 schools were eligible to participate. Due to closure or mergence with larger schools in the area nine 2010 schools were excluded. A recruitment letter was sent to the eligible schools, and followed up with a researcher phone call within a week. Of the 114 eligible schools, 74 (65%) participated in CSPPA 2018, 24 (21%) declined the invitation to participate and 16 (14%) could not be contacted and/or scheduled. Twelve convenience schools participated in CSPPA 2018. Convenience schools were schools who had not participated in CSPPA 2010 and were newly recruited for CSPPA 2018 to ensure representativeness across all year groups. (Woods et al., 2018).

For the NI CSPPA 2018 sample, an equivalence sample was drawn up to reflect the ROI sample. This sample consisted of 51 schools in total (20 primary, 31 secondary), of which 29 schools (9 primary, 20 secondary) were recruited to give an equivalence sample to the ROI sample (81% response rate). A recruitment letter was sent to these schools, and a researcher followed up with a phone call within seven days. In the case where a school declined to participate, a school with a similar stratification profile was approached to participate. (Woods et al., 2018).

In total, between the ROI and NI, 115 schools (48 primary, 67 secondary) participated in CSPPA 2018. Data collection for CSPPA 2018 was completed in spring 2018 and the first results were published in 2019. (Woods et al., 2018).

### 2.3 Data collection and study instruments

CSPPA provided 2 sources of information, firstly the researcher recorded (RR) school related factors and secondly the participant completed questionnaire. The CSPPA questionnaire was a multi-section, self-report instrument; developmentally appropriate and validated in this population. (Fahey et al., 2005, Woods et al., 2018, Woods, 2010) Questions for the primary (159 questions) and secondary students (245 questions) were different (Appendix A). Questionnaires were completed in school in small groups (n = 6–35) on tablets, supervised on a 10:1 ratio. Average time to completion was 35 min (range: 28–42 min). Passive parental consent and participant informed consent were obtained prior to questionnaire completion.

The questionnaire was validated and based on the Economic and Social Research Institute (ESRI) study, the Take PART study, and other published instruments. (Fahey et al., 2005, Woods et al., 2018, Woods et al., 2021, Woods, 2010) The questions chosen were developmentally appropriate, addressed the key areas of research interest and used categorical, Likert and ordinal scoring responses. This study focused on demographic, PA behaviour and correlates of PA questions (Appendix A). Family socioeconomic status was assessed using the Family Affluence Scale II, which stratifies individuals into low, medium or high family affluence. (Boyce et al., 2006, Woods et al., 2021) The questions selected to provide the data for this study are available in Appendix A.

### 2.4 Study design and ethical approval

This study is an observational, cross-sectional study. The Research Ethics Committee of the School of Public Health, Physiotherapy & Sports Science, University College Dublin approved this secondary analysis study. The Research Ethics Committees of the University of Limerick (EHSREC27_11_19), Dublin City University (DCUREC/2017/201) and Ulster University (REC/20/0005) granted ethical approval for CSPPA 2018. The anonymised CSPPA 2018 dataset was received as an IBM SPSS file from the Irish Social Science Data Archive (ISSDA). (Irish Social Science Data Archive, 2020).

### 2.5 Data management

Data management and analysis was completed using the statistical software SPSS version 27.0. Data was stored securely and password protected. Variables not relevant to this study were removed. Variables retained for use included variables that pertained to the commuting to school patterns and to the sociodemographic characteristics of the participants. A case report form (CRF) developed for this study illustrates the questions relating to each of the included variables (Appendix A). Included variables were largely categorical in nature. Some data manipulation was conducted prior to statistical analyses. Commuting method was recoded into 2 categories, active travel/transport to school (ATS) and passive travel/transport to school (PTS). Active commuters commute via bike or by foot. Passive commuters commute by car, bus or train i.e. by motorised transport. This was to allow for assessment of associations between ATS and variables. Where variables contained values for participants who refused to answer or answered, ‘Don’t Know’, these values were recoded as missing to exclude such values from statistical analysis. The full list of variables included in this study and their coding following data manipulation can be found in the data dictionary, Appendix B.

### 2.6 Data analysis

An initial descriptive analysis was conducted to illustrate the sociodemographic and commuting to school characteristics of the study population. These characteristics were described using numbers and percentages.

Murtagh et al. (2016) published longitudinal study research from The Growing Up in Ireland data 2008 to 2012 describing variables associated with ATS under four headings: Individual Level, Family Level, School Level and Neighbourhood Level. (Murtagh et al., 2016) To allow for consistency in Irish ATS data analysis, this study selected the same four headings to determine variables associated with ATS. In this study ATS was determined where a participant’s principal commute method to or from school was by non-motorised/self-propelled transport, most commonly by walking or cycling.

Potential predictor variables were included if they were previously shown to be associated with active commuting to school in either cross-sectional or longitudinal studies (Appendix C).

Univariate analysis was performed to identify associations between variables and commuting to school patterns. Pearson Chi Square test was used for univariate analysis of nominal categorical data. Following this, logistic regression was conducted. Independent variables included those significant on univariate analysis and results of logistic regression are presented as odds ratios (OR). A p-value of <0.05 was set for significance.

## 3 Results

### 3.1 Descriptive Statistics

The sociodemographic variables of the study participants (6,650) are shown in Table 2. Participants were ≥ 10 years old. The majority of the participants attended second level education (76.7%). One third of participating schools were located in NI with the remainder in ROI. The majority of pupils were classified as middle social class. Most schools were mixed gender and >66th percentile in pupil attendance size. Urban/rural school participation was close to even at both primary and secondary level. In ROI 16.7% of primary and 19.1% of secondary schools were designated DEIS.

**Table 2:** Sociodemographic Characteristics of Participants (CSSPA, Ireland, 2018).

	Primary School		Secondary School	
	N	Valid %*	N	Valid %*
**Participants**	1,548	23.3	5,102	76.7

**Gender**	1,548		5,100	
Male	712	46	2,364	46.4
Female	835	53.9	2,668	52.3
Other	1	0.1	68	1.3

**Age Categories**	1,548		5,100	
10 to 11	1,028	66.4	38	0.7
12 to 13	520	33.6	2,126	41.7
14 to 15			1,859	36.5
16 to 20			1,077	21.1

**Data Collection**	1,548		5,102	
NI	446	28.8	1,508	29.6
ROI	1,102	71.2	3,594	70.4

**Nationality**	1,548		5,100	
ROI	1,029	66.5	3,606	70.7
NI	226	14.6	621	12.2
Other	293	18.9	873	17.1

**SES**	1,543		4,989	
Low	342	22.2	1,022	20.5
Medium	873	56.6	2,937	58.9
High	328	21.3	1,030	20.6

**School Sex**	1,548		5,102	
Boys	81	5.2	842	16.5
Girls	216	14.0	1,353	26.5
Mixed	1,251	80.8	2,907	57.0

**DEIS/Non DEIS (ROI only)**	1,102		3,594	
DEIS School	184	16.7	326	9.1
Non DEIS School	918	83.3	3,268	90.9

**School Size**	1,548		5,102	
Small	190	12.3	534	10.5
Medium	458	29.6	1,137	22.3
Large	3,431	67.2	3,431	67.2

**School Location**	1,548		5,102	
Rural	739	47.7	2,956	57.9
Urban	809	52.3	2,146	42.1

SES: Socioeconomic status by family affluence scale (Boyce et al., 2006), ROI: Republic of Ireland, NI: Northern Ireland, DEIS: Delivering equality of opportunity in schools (Irish Department of Education, 2020).

* Valid % is the percent when missing data are excluded from the calculations.

Table 3 shows the commuting patterns of participants by primary and secondary school level. In total, 34.4% of participants commute to school actively, 36.4% primary and 27.7% secondary school pupils. Cycling was less common than walking for ATS. Primary school children commuting to school method was as follows: private transport (57%), walking (33.1%), public transport (6.7%), cycling (3.2%). Second level students recorded a higher proportion of public transport use (39.1%), with other commuting patterns being: private transport (30.4%), walking (28.4%), cycling (2.1%).

**Table 3:** Commuting Patterns of Participants (CSSPA, Ireland, 2018).

	Primary School		Secondary School	
	N	Valid %*	N	Valid %*
**N**	1,548	23.3	5,102	76.7

**Own a motorised vehicle?**	1,548		5,102	
No Vehicle		4.9		4.7
1		25.1		25.7
≥2		70.0		69.5

**Commute distance(km)**	Not assessed		5,100	
<1				17.0
1 to 5				36.1
5 to 10				23.6
>10				23.3

**Commute time (one way)**	1,548		5,102	
<5 min		28.5		9.5
5–10 mins		35.7		22.4
10–15 mins		18.7		23.1
15–20 mins		9.0		20.7
≥ 20 mins		8.1		24.2

**Principal commute type to/from school**	1,548		5,100	
Walk		33.1		28.4
Bike		3.2		2.1
Car		57.0		30.4
Bus		6.7		36.9
Train		0		2.2

**Already walk/cycle**	1,548		5,102	
Y		36.4		27.7
N		63.6		72.3

**Changes that would promote ATS:**	1,548		5,100	
Wider Footpaths		17.1		7.6
Safer Crossings		26.5		11.9
Less Traffic		21.1		12.1
Better Lighting		3.6		3.3
Cycle Lanes		19.2		9.0
Backpack Smaller/Lighter		19.6		25.5

**Specific barriers impeding change to ATS:**	1,548		5,100	
It is too far		6.3		35.5
Parents won’t allow me		15.6		8.5
No reason		9.4		8.1

**Past 7 days PA for ≥ 60mins**	1,548		5,100	
0		2.6		4.6
1		3.2		6.9
2		9.3		11.0
3		13.2		16.4
4		17.1		18.4
5		19.2		17.9
6		16.0		12.1
7		19.5		12.6

* Valid % is the percent when missing data are excluded from the calculations.

Participants who do not actively travel to school were asked what would encourage them to consider active travel. Primary school pupils would consider ATS if the following was possible in descending order of prevalence: safer road crossings (26.5%), less traffic (21.1%), smaller/lighter school backpack (19.6%), cycle lanes (19.2%), wider footpaths (17.1%), better lighting (3.6%). For secondary students the following were suggested: a smaller/lighter school backpack (25.5%), less traffic (12.1%), safer road crossings (11.9%), cycle lanes (9%), wider footpaths (7.6%), better lighting (3.3%).

### 3.2 Analytical Statistics

Table 4 shows the association of variables at the individual, family, school and neighbourhood level with ATS. Demographics associated with ATS investigation found; at an individual level age >12 years, ROI nationality and meeting daily PA guidelines were positively associated with ATS. Participant gender was not significantly associated with ATS.

**Table 4:** Demographics Associated with Active Travel to School (ATS) (CSSPA, Ireland, 2018).

Characteristics		ATS(Y) n	ATS(Y) Valid %*	ATS(N) n	ATS(N) Valid %*	p Value**
		2,289	34.4	4,359	65.6	

Individual Level						
**Age**						**0.003**
	≤12 years	774	33.8	1,321	30.3	
	>12 years	1,515	66.2	3,038	69.7	
**Gender**						**ns**
	Male	1,088	47.5	1,988	45.6	
	Female	1,176	51.4	2,327	53.4	
	Other	25	1.1	44	1	
**Nationality**						**<0.001**
	ROI	1,607	70.2	3,028	69.5	
	NI	192	8.4	655	15	
	Other	490	21.4	676	15.5	

**Meets PA Guidelines**		339	14.8	530	12.2	**0.002**

Family Level						
**Owns a motorised vehicle**						**<0.001**
	Yes	2,017	90.4	4,207	97.7	
	No	215	9.6	97	2.3	
**Family Affluence Level**						**<0.001**
	Low	543	24.4	821	19.1	
	Moderate	1,277	57.3	2,533	58.9	
	High	409	18.3	949	22.1	

School Level						
**School ROI/NI**						**<0.001**
	ROI	1,843	80.5	2,851	65.4	
	NI	446	19.5	1,508	34.6	
**School Gender**						**<0.001**
	Male	424	18.5	499	11.4	
	Female	663	29	906	20.8	
	Mixed	1,202	52.5	2,954	67.8	
**DEIS Status**						**<0.001**
	DEIS	325	17.6	183	6.4	
	Non DEIS	1,518	82.4	2,668	93.6	
**School Size**						**<0.001**
	Small	255	11.1	467	10.7	
	Medium	402	17.6	1,193	27.4	
	Large	1,632	71.3	2,699	61.9	
**School Location**						**<0.001**
	Rural	839	36.7	2,856	65.5	
	Urban	1,450	63.3	1,503	34.5	
**School Category**						**<0.001**
	Primary	634	27.7	914	21	
	Secondary	1,655	72.3	3,445	79	

Neighbourhood Level						
**Distance to School**						**<0.001**
	≤5 km	1,655	72.3	3,445	79	
	>5 km	634	27.7	914	21	

ATS(Y):Actively travel to school. ATS(N):Do not actively travel to school. ROI: Republic of Ireland, NI: Northern Ireland. Pearsons Chi-Square Test, ns: non-significant, **Significant p-value <0.05. *Valid % is the percent when missing data are excluded from the calculations.

Logistic regression is shown in Table 5. The strongest variables independently statistically significantly positively associated with ATS were; attending a ROI school (OR 2.27 95% CI 2.02–2.57), attending a non-DEIS school (OR 2.47 95 %CI 1.87–3.24) and attending an urban school (OR 3.96 95 %CI 3.41–4.59). The strongest factors independently statistically significantly negatively associated with ATS were; living in a family that owns a motorised vehicle (OR 0.27 95 %CI 0.19–0.39) and living > 5 km from school (OR 0.22 95 %CI 0.2–0.24).

**Table 5:** Logistic Regression: Factors Associated with Active Travel to School (ATS) (CSSPA, Ireland, 2018).

Variable		Unadjusted OR(95% CI)	Adjusted OR(95% CI)*	P Value
Individual Level				
Age ≥ 12 years		0.85(0.76–0.95)	0.97(0.93–1.01)	ns
Meets PA Guidelines		1.09(1.03–1.15)	1.26(1.08–1.46)	<0.05
Nationality-ROI		1.37(1.2–1.56)	1.09(1.02–1.16)	<0.05

Family Level				
Owns a Motorised Vehicle		0.22(0.17–0.28)	0.27(0.19–0.39)	<0.001
Low Relative Family Affluence		0.65(0.56–0.76)	0.96(0.86–1.06)	ns

School Level				
School in ROI		2.19(1.94–2.47)	2.27(2.01–2.57)	<0.001
Female School Gender		0.56(0.49–0.63)	0.96(0.87–1.05)	ns
Non DEIS Status		3.12(2.58–3.78)	2.47(1.87–3.24)	<0.001
Small School Size (<33rd percentile)		0.90(0.77–1.07)	0.68(0.60–0.77)	<0.001
Urban School		3.28(2.96–3.65)	3.96(3.41–4.59)	<0.001
Secondary School		0.87 (0.84–0.92)	0.69(0.62–0.78)	<0.001

Neighbourhood Level				
Distance from School (>5km)		0.67(0.06–0.08)	0.22(0.20–0.24)	<0.001

* OR and 95% CI are adjusted for all other variables in the table. ns: non-significant, significant p-value <0.05. ROI: Republic of Ireland.

## 4 Discussion

This study provides evidence of recent (2018) commuting to school patterns and factors associated with ATS in Ireland. In total, 34.4% of participants commute to school actively. ROI nationality, meeting PA guidelines, attending school in ROI, attending a non-DEIS school, and attending an urban school were each independently statistically significantly more likely to undertake ATS. Students living in a family with a car, attending secondary school, attending a small (<33rd percentile) sized school, living >5 km distance from school were significantly less likely to undertake ATS.

The proportion of primary and secondary pupils meeting the recommended 60 min of PA a week prior to survey was 19.5% and 12.6% respectively. Low levels of PA and sedentary lifestyles contribute to poor health, including obesity and reduced psychosocial health. (Tremblay et al., 2011) Children who actively commute to school by bike or foot have higher levels of PA, higher cardiorespiratory fitness, a healthier body composition and rate their health higher than passive commuters. (Chillón et al., 2011) Meeting PA guidelines is associated with physical health, psychsocial health, school performance and academic achievement. (Eime et al., 2013, Yang et al., 2023, James et al., 2023, López-Gil et al., 2020) Children and adolescents with less healthy lifestyles tend to exhibit poorer health in adulthood. (Lawrence et al., 2022) ATS may lead to healthy, environmentally sustainable and economical travel practices over the life course. (Carlin et al., 2017) If we do not act to intervene early in the youth population, physical inactivity will continue to perpetuate. (Lawrence et al., 2022).

An important finding was that 16% of primary school pupils who do not actively travel to school would consider doing so if their parents consented to their active commute. Intervention with parents of younger children can be explored to assess and address the concerns that are excluding their children’s ATS. A systematic review and *meta*-analysis of 84 articles by Jones et al. found that supervised walking to school buses and parental education were the most effective interventions to overcome parental resistance to ATS. (Jones et al., 2022) In Ireland there is support available to initiate and maintain such a service via local authority sport partnerships. (Sport Ireland. Local Authority Sport Partnerships, 2020) This resource could be highlighted to parents and schools, to ensure they are aware of this initiative. In the long term local authorities could consider funding and facilitating this service as they do with school traffic wardens, reducing the burden of volunteerism on parents with the state taking responsibility for operationalising such a health promoting service. (Citizens Information, 2022) Evaluation of such an initiative would be essential to determine if it could offer a potentially scalable solution to increase ATS participation nationally and internationally.

If their school backpack was lighter, 1 in 4 second level participants who do not ATS would consider commuting actively, similar to New Zealand research findings of 31%. (Mandic et al., 2021) Collaboration between school teachers and students to find solutions to reduce the weight of school backpacks is a low cost intervention that is quickly actionable and holds potentially to reduce this barrier to ATS participation amongst this adolescent group.

Greater distance to school is a factor that is independently negatively associated with ATS which is reflected widely in the literature, including Irish research by Murtagh et al. (2016) who suggest this should be considered in future planning of new school development location. (Murtagh et al., 2016) In addition, ensuring adequate public transport service networks is essential when planning school location and commuting options. The majority of participants’ families owned a motorised vehicle (95%) and motorised vehicle ownership was associated with passive travel to school. If more public transport options were available for commuting to school this could reduce the barrier that car ownership poses to ATS, as well as support other societal benefits, since public transport use can involve an element of PA and can reduce traffic congestion and fuel emission during school commute times. (Tønnesen et al., 2021, Institute of Transport Economics, Norwegian Centre of Transport Research, 2017, Naess, 2012).

Murtagh et al. (2016) reported significant associations with gender and participant PA level at an individual level, socioeconomic status at a family level, school size at a school level and distance to school at a neighbourhood level. (Murtagh et al., 2016) Parallel significant associations with ATS at an individual level were meeting PA guidelines, at a family level were socioeconomic status and at a school level were attending a school >33rd percentile in size, attending an urban school and attending a primary school. At a neighbourhood level living ≤5 km from school was significantly associated with ATS in both studies. (Murtagh et al., 2016).

Further research is required to translate the evidence base for the most optimal and most cost effective interventions to increase ATS in Ireland using the associations established in this study. This study’s findings can direct further research to determine how to; appease the anxiety of parents concerning ATS, optimally pilot the introduction of supervised walking/cycling to school buses, optimally increase ATS participation in rural, small sized, disadvantaged schools, secondary schools, for students living in a family that owns a car and/or live >5 km from school.

At present, a systems approach, is recommended to increase PA worldwide. (Murphy et al., 2021) This research and previous research can inform a multi-strategic systems approach to increasing ATS and result in increased PA amongst youth in Ireland. (Murtagh et al., 2016, Woods et al., 2021) Such an approach could include public policy design to support ATS, financial support for purchase and hire of cycling equipment where required, school support, parental support, culture shift away from private transport, urban and rural planning, public transport service planning. A coordinated systems approach with multi-sector investment could design and deliver an environment that supports and facilitates ATS. (Woods et al., 2021) Long term infrastructure policy and incentives to enable and empower ATS must be considered in line with the systems approach. (Murphy et al., 2021) Ensuring safe walking and cycling routes to schools is required. This study highlighted that those who do not actively commute to school (65.6%) would consider ATS if there was one or more of the following; wider footpaths, safer crossings, less traffic, better lighting and segregated cycle lanes. In March 2021 the Irish government announced €70M state funding to support active commuting safe segregated networks. (Oireachtas, 2022) Further CSPPA iterations could assess if current and future infrastructure modifications influence ATS.

There are a number of strengths to this study. CSPPA 2018 is a large (115 schools and 6650 students participated), population representative dataset. The multi-stage probability sampling strategy utilised ensured the sample was population representative in terms of nationality, socioeconomic status and urban/rural residence. (CSO, 2016, Organisation for Economic Co-operation and Development, 2020) A validated tool to design the study questionnaire was utilised and response rate was 81%. (Irish Social Science Data Archive, 2020) This was the first observational research to include both ROI and NI schools creating an all-island approach to research, which can be carried forward into the actions taken based on the study findings.

There are also a number of limitations. The study is a cross-sectional design, thus it is not possible to draw causal inferences from the results. Schools elected to participate in the study when invited, introducing self-selection bias since the participating schools may be more dedicated to sport and PA than the schools that declined to participate. The CSPPA study is carried out via questionnaire with the potential to introduce subjective self-report bias. The self-report nature may introduce bias in particular since the pupils are young at ≥10 years. There was no qualitative element to this study and participants were confined to selecting a response from a pre-determined list, thus answers are not fully reflective of participants own ideas or thoughts. While the voices of children and youth are invaluable, youth may not have volitional control of their behaviours. This research did not investigate the input of parents/guardians. The voice of parents/guardians, who are also voters, financial stakeholders and responsible for their children's health may unveil barriers that can be addressed at the parent level that influence child ATS behaviour, nonetheless the findings of this study have important public health implications for the complete island of Ireland.

## 5 Conclusions

This study described the commuting to school patterns of children and adolescents in Ireland and examined the association of factors with ATS. An understanding of ATS associations is essential to inform public health planning and action towards interventions to increase ATS participation and allow for the discussed multiple benefits of ATS to be realised by this population. (Murtagh et al., 2016).

## Declaration of Competing Interest

The authors declare that they have no known competing financial interests or personal relationships that could have appeared to influence the work reported in this paper.

## Figures and Tables

**Table:** 

Project Title: Child and Adolescent Patterns of Commuting to School				
Investigators: Dr. C. Power, Prof. P. Fitzpatrick				
Q/RR	Category	Question	Answer Options	Researcher Comments
		Administrative Participant Data		
RR		Participant Study ID Number	Assigned	
RR		School Code	Assigned	
RR	1° or 2°	Is the participant in a 1° or 2° school?	Primary (1°)/Secondary (2°)	2 categories

Participant Questions				
Demographic Data Section				
3	Gender	Are you a boy, girl or other? Please tick.	1° Boy/Girl/Other2° Male/Female/Other	3 categories
4	Age	What age are you? Please tick.	10/11/12/13/14/15/16/17/18/19/20	11 categories coded into 2:≤12 years and >12 years
5	Nationality	What nationality are you? Please tick.	17 categories of nationality offered including ROI, NI and Other	17 categories coded into 3:ROI, NI and Other
83	Area of Residence	Where do you live? Please tick.	City (>70,000 inhabitants)Suburb(<70,000 inhabitants) Town (<20,000 inhabitants)Village (<3,000 inhabitants)	4 categories
171	Family Vehicle Ownership	Does your family own a car, van or truck?	No/1/≥2	3 categories used to assess Family Affluence Score II also
21	Level of PA	Over the past week how many days did you exercise for ≥ 60 min?	0/1/2/3/4/5/6/7	8 categories
170	SES	How many computers does your family have?	0/1, 2, >2	3 categories used to assess Family Affluence Score II*
171	SES	Does your family own a car/van/truck?	No,1, ≥2	3 categories used to assess Family Affluence Score II*
172	SES	How many times did you travel abroad on holiday last year?	0/1/2/>2	4 categories used to assess Family Affluence Score II*
173	SES	Do you have your own bedroom?	Yes/No	2 categories used to assess Family Affluence Score II*
174	SES	Does your home have a dishwasher?	Yes/No	2 categories used to assess Family Affluence Score II*
175	SES	How many bathrooms in your home?	0/1, ≥2	2 categories used to assess Family Affluence Score II*

Commuting Questions				
23	Distance to school	What distance is your journey to school? (km)	Not assessed in 1° students<1 km1–5 km5–10 km≥10 km	4 categories
24	Commute Time	How long does it usually take to travel to school (one way)?	<5 mins5–10 mins10–15 mins15–20 mins≥20 mins	5 categories
25	Commute Type	How do you usually travel to school?	By FootBicycleCarBusTrain	5 categories
26	Commute Type	How do you usually travel home from school?	By FootBicycleCarBusTrain	5 categories
27	ATS Barriers	What might encourage you to walk/cycle to school? (Tick as many options as appropriate)	I already walk/cycleWider footpathsCycle lanesBetter lightingLess trafficSafer places to cross the roadNothing; it is too farNothing; my parents will not allow itNothingIf my schoolbag was lighter/smaller	10 categories
28	Commute Type	How do you mostly travel to and from school?	By FootBicycleCarBusTrain	5 categories

Administrative School Data				
RR		School Code	Assigned	
RR	Location	ROI or NI?	ROI/NI	2 categories
RR	1° or 2°	1° or 2° school?	1°/2° (Secondary)	2 categories

	School Demographics (Answered by School Principals recorded by researchers)			
RR	Gender	School Gender	MaleFemaleMixed	3 categories
RR	Deis/Non Deis	Is this school a DEIS or a non-DEIS school?	DEISNon DEISNA(NI Schools)	3 categories
RR	Size	Tick the school size category**	LargeMediumSmall	3 categories
RR	Location	School Location***	Urban/Rural	2 categories

**Table:** 

Project Title: Child and Adolescent Patterns of Commuting to School						
Investigators: Dr. C. Power, Prof. P. Fitzpatrick						
Q/RR	Abbr.	Variable Definition	Variable Type		Level of Measurement	Coding
			Qualitative/Quantitative	Categorical/Discrete/Continuous		
RR	PC	Participant Study ID Number	Quantitative	Discrete	Nominal	Enter as givenMissing = 999
RR	SC	School Code	Quantitative	Discrete	Nominal	Enter as givenMissing = 999
RR	PSec	Is the participant in a primary (1°) or secondary (2°) school?	Qualitative	Categorical	Ordinal	1°=1,2°=1, Missing = 999

Participant Questions						
Demographic Data Section						
3	Gender	Are you a boy, girl or other? Please tick.	Qualitative	Categorical	Nominal	Male = 1,Female:2,Other = 3, Missing = 999
4	Age	What age are you? Please tick.	Quantitative	Discrete	Interval	Entered as given(10/11/12/13/14/15/16/17/18/19/20), Missing:9
4	Age12	New Variable	Quantitative	Categorical	Ordinal	Coded into ≤ 12 years = 1,>12 years = 2, Missing = 999
5	Nat	What nationality are you? Please tick.	Qualitative	Categorical	Nominal	17 categories of nationality numbered 1–17 as per questionnaire listing, Missing = 999
5	Nat3	New Variable	Qualitative	Categorical	Nominal	17 categories coded into ROI = 1, NI = 2Other = 3, Missing = 999
83	AofR	Where do you live? Please tick.	Qualitative	Categorical	Nominal	City = 1,Suburb = 2,Town = 3, Village = 4, Missing = 999
171	OwnVehicle	Does your family own a car, van or truck?	Qualitative and Quantitative	Categorical	Ordinal	No = 0, 1 = 1, ≥2 = 2, Missing = 999
21	PA	Over the past week how many days did you exercise for ≥ 60 min?	Quantitative	Discrete	Ordinal	Entered as given(0/1/2/3/4/5/6/7) Missing:9
21	MeetsPA	New VariableMeets PA guidelines (Y/N). Y-7 days, N-0–6 days)	Qualitative	Categorical	Nominal	Yes = 1,No = 2, Missing:9
170	SESComp *	How many computers does your family have?	Quantitative	Categorical	Ordinal	0/1 = 1,2 = 2,>2 = 3,Missing:9
171	OwnVehicle	Does your family own a car, van or truck?	Qualitative and Quantitative	Categorical	Ordinal	No = 0, 1 = 1, ≥2 = 2, Missing = 999
172	SESTravel *	How many times did you travel abroad on holiday last year?	Quantitative	Discrete	Ordinal	0 = 0, 1 = 1, 2 = 2,>2 = 3, Missing = 999
173	SESBedR *	Do you have your own bedroom?	Qualitative	Categorical	Ordinal	Yes = 1, No = 2 Missing = 999
174	SESDW *	Does your home have a dishwasher?	Qualitative	Categorical	Ordinal	Yes = 1, No = 2 Missing = 999
175	SESBathR *	How many bathrooms in your home?	Quantitative	Discrete	Ordinal	0/1 = 1, ≥2 = 2, Missing = 999
	FamilyA*	Family Affluence II	Qualitative	Categorical	Ordinal	Low = 1, Medium = 2, High = 3, Missing = 999

Commuting Questions						
23	D2school	What distance is your journey to school? (km)	Quantitative	Discrete	Ordinal	<1km = 1,1–5 km = 2,5–10 km = 3,≥10 km = 4,Missing = 999
	D2School5km	New Variable	Quantitative	Discrete	Nominal	≤5km = 1,>5km = 2,Missing = 999
24	CommuteTime	How long does it usually take to travel to school (one way)?	Quantitative	Discrete	Ordinal	<5mins = 1,5-10mins = 2,10–15 mins = 3,15-20mins = 4,≥20 mins = 5, Missing = 999
25	ComType2S	How do you usually travel to school?	Qualitative	Categorical	Nominal	By Foot = 1,Bicycle = 2,Car = 3,Bus = 4,Train = 5,Missing = 999
26	ComTypeFS	How do you usually travel home from school?	Qualitative	Categorical	Nominal	By Foot = 1,Bicycle = 2,Car = 3,Bus = 4,Train = 5,Missing = 999
	ATS	New VariableATS = Walk/Bike	Qualitative	Categorical	Nominal	Yes = 1,No = 2, Missing = 999
27	ATSBarriers	What might encourage you to walk/cycle to school? (Tick as many options as appropriate)	Qualitative	Categorical	Nominal	I already walk/cycle = 1, Wider footpaths = 2,Cycle lanes = 3,Better lighting = 4,Less traffic = 5,Safer places to cross the road = 6,Nothing;it is too far = 7,Nothing;my parents will not allow it = 8,Nothing = 9If my schoolbag was lighter/smaller = 10,Missing = 999
28	ComTypeMC	How do you mostly travel to and from school?	Qualitative	Categorical	Nominal	By Foot = 1,Bicycle = 2,Car = 3,Bus = 4,Train = 5,Missing = 999

Administrative School Data						
RR	SC	School Code	Quantitative	Discrete	Nominal	Enter as givenMissing = 999
RR	LocSc	ROI or NI?	Qualitative	Categorical	Nominal	ROI = 1,NI = 2, Missing = 999
RR	PSec	Is the participant in a 1° or 2° school?	Qualitative	Categorical	Ordinal	1°=1,2°=1, Missing = 999

School Demographics (Answered by School Principals recorded by researchers)						
RR	SchoolGender	School Gender	Qualitative	Categorical	Nominal	Male = 1,Female = 2,Mixed = 3,Missing = 999
RR	DND	Is this school a DEIS or a non-DEIS school?	Qualitative	Categorical	Nominal	DEIS = 1,Non DEIS = 2,NA(NI Schools) = 3, Missing = 999
RR	SchoolSize	Tick the school size category**	Qualitative	Categorical	Ordinal	Large = 1,Medium = 2,Small = 3, Missing = 999
RR	SchoolLoc	School Location***	Qualitative	Categorical	Nominal	Urban = 1,Rural = 2, Missing = 999

**Table:** 

Variables	Longitudinal Studies	Cross-Sectional Studies	Other
Individual Level			
Age	Murtagh et al. (2016)	White et al. (2021), Mandic et al. (2014), D’Haese et al. (2011)	SR: Tremblay et al. (2011)
Gender	Murtagh et al. (2016)	Woods et al. (2021), Mandic et al. (2014), Davison et al. (2008), Cooper et al. (2005)	
Nationality	Murtagh et al. (2016)		CSO (2016), Pont et al. (2009) SR of Cross-sectional Studies
Meets PA Guidelines	Murtagh et al. (2016)	Faulkner et al. (2009), Cooper et al. (2005)	
Family Level			
Motorised Vehicle Ownership	Clark et al. (2016)	Kweon et al. (2006)	Pont et al. (2009) SR of Cross-sectional Studies
Family Affluence Level	Murtagh et al. (2016), Panter et al. (2011)	Woods et al. (2021), Davison et al. (2008)	Pont et al. (2009) SR of Cross-sectional Studies
School Level			
School Location (ROI or NI)			Pont et al. (2009) SR of Cross-sectional Studies
School Gender	Murtagh et al. (2016)	Woods et al. (2021)	
DEIS or Non DEIS School		Woods et al. (2021)	
School Enrolment Size	Murtagh et al. (2016)	Davison et al. (2008)	
Location: Rural/Urban	Murtagh et al. (2016)	Panter et al. (2008)	CSO, 2016, CSO, 2019
Category: Primary/Secondary	Murtagh et al. (2016)	Daniels (2014)	
Neighbourhood Level			
Distance to School	Murtagh et al. (2016), Chillón et al. (2011), Panter et al. (2011), Larouche et al. (2013)	D’Haese et al. (2011), Ziviani et al. (2004), Nelson et al. (2008), Panter et al. (2008), Kweon et al. (2006)	Pont et al. (2009) SR of Cross-sectional Studies

## Data Availability

The authors do not have permission to share data.

## Appendix A This study case report form

ROI: Republic of Ireland, NI: Northern Ireland. DEIS (Delivering Equality of Opportunity In Schools) school is a school classified as disadvantaged under criteria which qualifies for additional supports from Dept. of Education in an effort to promote equity in education. (Irish Department of Education, 2020)

*Assessing Relative Family Affluence Score II (Boyce et al., 2006)

** School Size Category.

Size: small, medium or large (based on total number of pupils). For the Republic of Ireland, school sizes were determined by creating tertiles using all primary or post primary schools (tertile 1 = small, tertile 2 = medium and tertile 3 = large). Small: <33rd percentile, medium: 33rd – 66th percentile, large: >66th percentile. For Northern Ireland, the school size was supplied by the Department of Education Northern Ireland.

*** School location: urban or rural (categorised by population density).

## Appendix B This study data dictionary

NI;Northern Ireland, ROI: Republic of Ireland. SES: Socioeconomic Status. DEIS (Delivering Equality of Opportunity In Schools) school is a school classified as disadvantaged under criteria which qualifies for additional supports from Dept. of Education in an effort to promote equity in education (Irish Department of Education, 2020).

**Used to assess Family Affluence Score II, Assessing Relative Family Affluence Score II, (Boyce et al., 2006)

** School Size Category.

Size: small, medium or large (based on total number of pupils). For the Republic of Ireland, school sizes were determined by creating tertiles using all primary or post primary schools (tertile 1 = small, tertile 2 = medium and tertile 3 = large). Small: <33rd percentile, medium: 33rd – 66th percentile, large: >66th percentile. For Northern Ireland, the school size was supplied by the Department of Education Northern Ireland.

*** School location: urban or rural (categorised by population density).

## Appendix C Variables associated with active travel to school (ATS)

DEIS (Delivering Equality of Opportunity in Schools). DEIS school is a school classified as disadvantaged under criteria which qualifies for additional supports from Dept. of Education in an effort to promote equity in education. (Irish Department of Education, 2020)
